# Macrophage Polarization and Functions in Pathogenesis of Chronic Obstructive Pulmonary Disease

**DOI:** 10.3390/ijms25115631

**Published:** 2024-05-22

**Authors:** Gun-Dong Kim, Eun Yeong Lim, Hee Soon Shin

**Affiliations:** 1 Division of Food Functionality Research, Korea Food Research Institute (KFRI), Wanju 55365, Republic of Korea; kgd@kfri.re.kr (G.-D.K.); l.eunyeong@kfri.re.kr (E.Y.L.); 2 Department of Food Biotechnology, Korea University of Science and Technology (UST), Daejeon 34113, Republic of Korea

**Keywords:** chronic obstructive pulmonary disease, macrophage, polarization, inflammation, reactive oxygen species

## Abstract

Chronic obstructive pulmonary disease (COPD), the major leading cause of mortality worldwide, is a progressive and irreversible respiratory condition characterized by peripheral airway and lung parenchymal inflammation, accompanied by fibrosis, emphysema, and airflow limitation, and has multiple etiologies, including genetic variance, air pollution, and repetitive exposure to harmful substances. However, the precise mechanisms underlying the pathogenesis of COPD have not been identified. Recent multiomics-based evidence suggests that the plasticity of alveolar macrophages contributes to the onset and progression of COPD through the coordinated modulation of numerous transcription factors. Therefore, this review focuses on understanding the mechanisms and functions of macrophage polarization that regulate lung homeostasis in COPD. These findings may provide a better insight into the distinct role of macrophages in COPD pathogenesis and perspective for developing novel therapeutic strategies targeting macrophage polarization.

## 1. Introduction

COPD is a chronic inflammatory lung condition characterized by irreversible airflow limitation caused by a complex interplay of small airway disease and lung parenchymal destruction in long-term progression [1]. Chronic inflammation induces structural changes, such as airway fibrosis in the small airways and the destruction of the alveoli in the pulmonary parenchyma, leading to disconnection between the alveoli and small airways and reduced lung elasticity, and resultant airflow limitation characterized by narrowing or failure to open the airways [2]. Factors involved in the onset and progression of COPD can be broadly categorized into the host and external factors. Host factors include genetic variance, aging, gender, lung growth, and airway hypersensitivity, while external factors involve smoking, air pollution, and socioeconomic factors [1,3,4,5,6]. Among these factors, smoking is the most well-known, with smokers exhibiting a higher prevalence of respiratory symptoms than non-smokers, along with higher mortality rates and a declining annual forced expiratory volume in 1 s (FEV1) [7]. According to a report by the American Thoracic Society, occupational exposure to hazardous substances is a well-recognized risk factor. Even after adjusting for smoking and age, the duration of employment for coal miners, quarry workers, tunnel workers, and concrete workers has been reported to be associated with progressive declines in lung function (FEV1: −7 to 8 mL/year) [8]. Ambient air pollution also plays an important role in reducing lung function, maturation, and development. In particular, children with a clinically declined FEV1 exhibited a positive correlation with the exposure levels of particulate matter (PM) 2.5. The estimated low FEV1 proportion in places with the highest and lowest levels of exposure to PM2.5 across the twelve communities was 7.9% and 1.6%, approximately five-fold changes, respectively [9]. Additionally, long-term exposure to PM has been reported to be associated with the development of COPD and deterioration in lung function. For every 7 μg/m^3^ increase in the PM concentration over five years, there is a 5.1% decrease in FEV1 and a 3.7% decrease in the forced vital capacity (FVC), while the incidence of COPD increases by 33% [10]. In 2019, the disability-adjusted life years (DALY) attributable to COPD were found to be dominated by smoking (46%), followed by ambient PM pollution (20.7%) and occupational exposure to PM, gases, and fumes (15.6%) [11]. According to the World Health Organization (WHO), approximately 212.3 million people worldwide were diagnosed with COPD in 2019 [12]. Furthermore, research conducted as part of the Global Burden of Disease showed that approximately 480 million patients with COPD had a worldwide prevalence of 10.6% in 2020 [13]. Similarly, in studies using the Global Initiative for the Chronic Obstructive Lung Disease (GOLD) standard of FEV1/FVC < 0.7, a prevalence of 10.9% to 12% is generally predicted, suggesting that there will be 300 to 400 million patients with COPD worldwide from 2018 to 2019 [13]. The morbidity of COPD is influenced by overlapping chronic diseases such as cardiovascular disease, musculoskeletal disease, lung cancer, and diabetes. These comorbidities affect the patient’s health status and make COPD treatment difficult [1]. In the United States, more than 5% of adults have COPD, which is ranked 12th among all diseases. According to a WHO assessment, approximately 65 million patients have moderate-to-severe COPD. In 2019, COPD was the third leading cause of death globally, with 3.28 million deaths attributed to COPD, representing 74.4 million DALYs [11]. COPD-attributable mortality was higher in males compared to females, with 1.88 million deaths in males, 1.39 million deaths in females, and 42.05 million DALYs in males compared to 32.37 million DALYs in females [11]. Additionally, more than 90% of COPD-attributable deaths occur in low- or middle-income countries. From 2020 to 2050, the number of COPD cases is estimated to increase to 592 million, accounting for 9.5% of the total population; consequently, co-morbidities are also expected to increase in parallel with this trend [13]. Owing to the global trend of the increasing prevalence and mortality rates of COPD, there is an imperative need for research on the pathology, etiology, and pathophysiology of COPD to facilitate prevention and treatment strategies.

**Figure 1:**
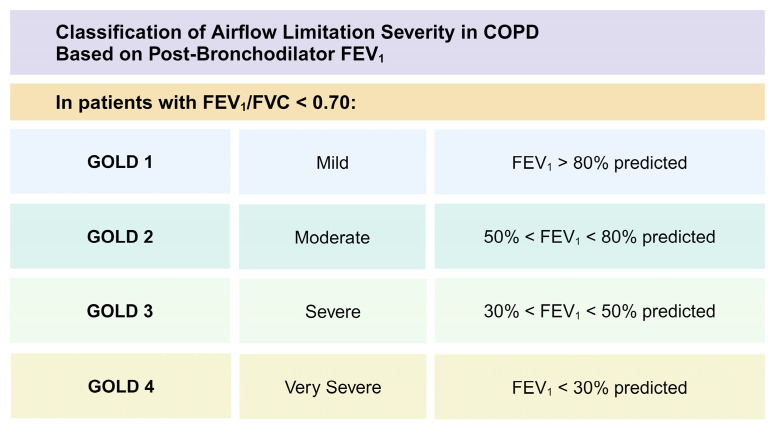
The GOLD guidelines classify patients into four different categories. COPD: chronic obstructive pulmonary disease; FEV1: forced expiratory volume in 1 s; FVC: forced vital capacity. Created with BioRender.com. Last accessed on 10 April 2024.

In 2001, the National Heart, Lung, and Blood Institute in the United States, in collaboration with the WHO, led the GOLD committee to hold a consensus workshop on the diagnosis, prevention, and treatment of COPD and published a summary report on the GOLD guidelines for the first time [14]. Previously, the GOLD guidelines classified patients into four categories based on the level of airflow limitation: GOLD 1 (mild), GOLD 2 (moderate), GOLD 3 (severe), and GOLD 4 (very severe) [14]. Detailed information regarding the GOLD classification and FEV1 values is represented in Figure 1. FEV1 is based on manipulation and may not always correlate with clinically relevant outcomes.

**Figure 2:**
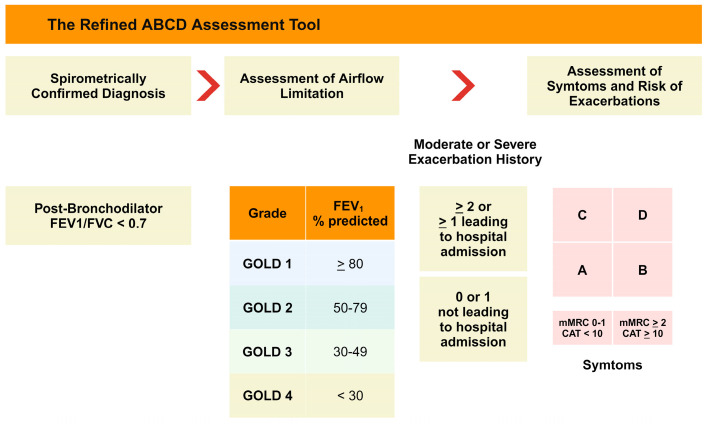
The refined ABCD assessment tool of GOLD guidelines. ABCD: Group A: low risk/low symptoms, Group B: low risk/high symptoms, Group C: high risk/low symptoms, Group D: high risk/high symptoms; CAT: COPD Assessment Test; COPD: chronic obstructive pulmonary disease; FEV1: forced expiratory volume in 1 s; FVC: forced vital capacity; mMRC: modified British Medical Research Council. Created with BioRender.com. Last accessed on 10 April 2024.

Therefore, in the 2011 GOLD strategy, revised guidelines were introduced to address these issues, incorporating FEV1, the modified Medical Research Council (mMRC), the dyspnea scale or COPD Assessment Test (CAT) score, and exacerbation history [15]. The mMRC questionnaire and CAT are the two most widely used methods for evaluating COPD symptoms. This phenotypic classification better reflects the complexity of COPD than FEV1 alone. The revised GOLD group classification is shown in Figure 2.

## 2. Pathophysiology of COPD

The pathological changes in COPD are distributed across the small airways, lung parenchyma, and pulmonary vasculature and are characterized by the destruction of the lung parenchyma due to chronic inflammation and injury [2]. Pathological changes resulting from inflammation in the airway lumen and peripheral airway stenosis predominantly manifest as decreased FEV1, whereas parenchymal damage due to emphysema primarily leads to decreased FEV1 and the dysregulation of the gas exchange [2]. Additionally, obstruction of the small airways is developed. To prevent and provide appropriate treatment for COPD, it is essential to understand its pathophysiology and etiology.

### 2.1. Airflow Limitation

The most critical pathophysiology of COPD is the chronic and irreversible airflow limitation, characterized by increased airway resistance and decreased lung compliance [14]. Airway resistance is normally within the range of 0 to 1 cm H_2_O·L^−1^·s, whereas in COPD, it is reported to increase to 5 to 15 cm H_2_O·L^−1^·s or higher [16,17]. This increase in airway resistance primarily arises from an increase in resistance in the small airways, typically 2 mm or smaller, and is considered the most important mechanism underlying airflow limitation in COPD [18,19]. The airflow limitation is associated with a decreased FEV1 and FEV1/FVC ratio, inflammation, fibrosis, and mucus production within the small airways [2,20,21]. Air trapping due to small airway obstruction worsens progressively, leading to excessive lung expansion. Lung hyperinflation decreases the inspiratory capacity during exercise and increases the functional residual capacity, ultimately resulting in dynamic hyperinflation [22]. This can exacerbate breathlessness and induce limitations in exercise. Moreover, hyperinflation influences the contractility of respiratory muscles and activates local inflammatory mediators [22,23].

### 2.2. Small Airway Obstruction

Airway obstruction is predominantly observed in small airways, typically between the branching 4th and 14th airways [18,24]. In normal lungs, peripheral airway resistance accounts for approximately 25% of the total lower airway resistance compared to that in patients with emphysema and has been reported to range from 63% to 93% [18,19]. According to studies examining the histopathology of small airways in lung tissues from patients with COPD involved across all stages of the GOLD classification, it has been revealed that as the GOLD stage progresses, there is an infiltration of inflammatory cells forming lymphoid follicles, leading to the thickening of the airway walls and increased inflammatory mucoid exudate within the small airways [19,21]. Small airway obstruction develops from both reversible factors, including airway inflammation, excessive mucus secretion, and smooth muscle contraction due to airway hyper-responsiveness, and irreversible factors, such as increased smooth muscle and connective tissue in the small airways and fibrosis around the small airways, leading to an irreversible reduction in the luminal diameter [25,26].

### 2.3. Emphysema

Emphysema is defined as an abnormal expansion of the air space located at the end of the terminal bronchiole and tissue destruction unrelated to fibrosis progression [27,28]. Emphysematous destruction of the lungs results in a decrease in the maximal expiratory flow owing to diminished elastic recoil [29]. Emphysema is classified into centrilobular, panacinar, and paraseptal types. Centrilobular emphysema is most closely associated with smoking and is known to exhibit more severe small airway obstruction than panacinar emphysema [30,31]. Panacinar emphysema is associated with an α1-antitrypsin deficiency and typically begins in the lower lobes of the lungs, extending across all lobes, with a tendency for tissue destruction [30,32]. Although there is an association between smoking and emphysema, it is not always strong. Only 40% of heavy smokers develop emphysema, and only 15% develop airflow limitation, reflecting the fact that individuals with emphysema may still maintain normal lung function [2,33]. Emphysema is primarily known to arise from an imbalance in proteolytic enzymes, and a recent study showing emphysematous lesions induced by enzyme accumulation, such as neutrophil elastase, in the lung tissue supports this association [34,35].

### 2.4. Chronic Bronchitis

Chronic bronchitis is associated with various clinical outcomes including an accelerated decline in lung function, an increased risk of airflow obstruction in smokers, a tendency for decreased respiratory infections, an increased frequency of exacerbations, and worsened overall mortality [36]. The prevalence of chronic bronchitis appears to be higher in patients with COPD, affecting approximately 14–74% of patients with COPD [37,38]. Chronic bronchitis is primarily induced by the overproduction of mucus from goblet cells in the epithelium of central airways with an internal diameter of 4 mm or more, which then extends into mucus-secreting glands [2,39]. The hypertrophy of mucus glands in the bronchi in chronic bronchitis supports this outcome. Additionally, the impairment of the mucociliary clearance function, destruction of the epithelial barrier by the innate host defense system, and unbridled immune responses due to excessive infiltration of inflammatory cells exacerbate the inflammatory response [36,40]. In an observational study conducted over 8–10 years in 9435 Danish individuals, chronic excessive mucus secretion was significantly associated with an accelerated decline in FEV1 and an increased risk of COPD-related hospitalization [41].

### 2.5. Airway Mucus Hyperproduction

Excessive mucus secretion chronically induces a productive cough, which is a characteristic symptom of chronic bronchitis. However, it is not solely associated with airflow limitation. The excessive mucus production observed in COPD pathogenesis results from an increase in the goblet cell number due to inflammatory mediators, such as leukotrienes, proteolytic enzymes, and neuropeptides, and the enlargement of the mucus-secreting glands [42,43]. Chronic airway irritation from cigarette smoke (CS) or harmful substances induces an increase in the number of goblet cells and enlargement of submucosal glands, leading to mucus hyperproduction, which can subsequently induce small airway obstruction and ventilation dysfunction in the bronchioles [44,45]. Proteolytic enzymes promote excessive mucus production, primarily through the activation of epidermal growth factor receptors. Furthermore, epithelial cells containing cilia induce squamous metaplasia, leading to the dysfunction of mucociliary clearance through ciliary motility [46,47].

### 2.6. Impairment of Gas Exchange

The most significant gas exchange impairment in COPD results from an imbalance in pulmonary ventilation–perfusion [48,49]. As the disease progresses, peripheral airway wall injuries and reduced ventilation due to the loss of lung elastic recoil in emphysema lead to a ventilation–perfusion imbalance along with overall ventilation heterogeneity and a loss of pulmonary capillaries, ultimately resulting in hypoxemia [48,50]. Additionally, gas exchange abnormalities worsen, leading to increased work of breathing due to severe airway obstruction, respiratory muscle abnormalities, and hyperinflation, resulting in hypoventilation [48]. This hypoventilation leads to carbon dioxide retention, and chronic hypercapnia typically reflects dysfunction of inspiratory muscles and hypoventilation at the cellular level [48].

## 3. Etiology of COPD

**Figure 3:**
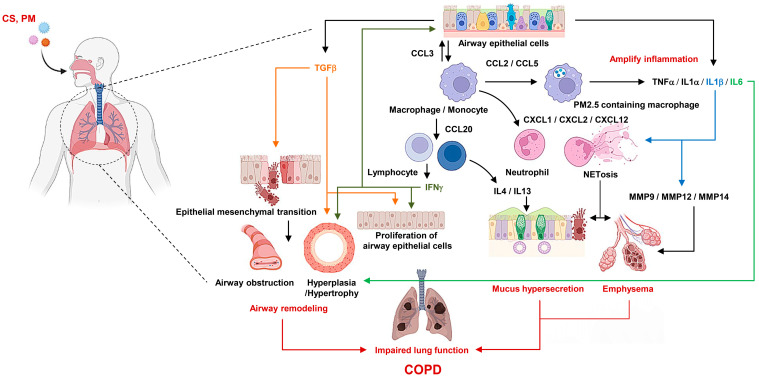
Overview of the pathogenesis of COPD. Pathological changes in COPD are distributed throughout small airways, lung parenchyma, and pulmonary vasculature and are characterized by chronic inflammation and destruction of lung parenchyma mediated by inflammatory cells such as airway epithelial cells, neutrophils, and macrophages. Parenchymal damage due to inflammation of the airway lumen, peripheral airway stenosis, and emphysema causes airflow limitation, impaired gas exchange, small airway obstruction, and decline of lung function. Created with BioRender.com. Last accessed on 10 April 2024.

COPD is characterized by chronic pathological changes in the airways, lung parenchyma, and pulmonary vasculature [1]. In each region of the lung, specific cell-mediated inflammatory responses and structural alterations occur in the airways due to repeated injury and repair processes. Disease severity is associated with increased inflammation and structural changes. The major factors in COPD pathogenesis include oxidative stress within the lungs, an imbalance between proteolytic and anti-proteolytic enzymes, and inflammatory responses. These etiological factors can arise from environmental or genetic factors (Figure 3).

### 3.1. Oxidative Stress

Among the many factors that induce oxidative stress, smoking is the most directly associated with COPD [1]. CS consists of over 4700 chemicals, including nicotine, tar, acrolein, and polyaromatic aromatic hydrocarbons, along with various free radicals and oxidants [51,52]. It is well known that cigarette smoke activates neutrophils and macrophages, leading to the generation of reactive oxygen species, nitric oxide, and 8-isoprostane, as well as various free radicals such as O_2_^−^ and H_2_O_2_ [53]. Extracellular matrix (ECM) components such as elastin are also known to be damaged by smoking and oxidative stress, leading to the impairment of their synthesis and repair processes, indicating that oxidative stress can contribute to the development of emphysema [54]. Epithelial cells of the lungs are also damaged by oxidative stress, leading to increased cell permeability. In epithelial cells, the antioxidant activity of glutathione (GSH), which is present inside and outside the cells, is crucial [55,56]. However, in heavy smokers, the concentration of GSH in the bronchoalveolar lavage fluid (BALF) is insufficient to neutralize excessive oxidative stress, and it is known to be depleted in proportion to the duration and amount of exposure to smoking, leading to an imbalance between oxidants and antioxidants [57]. Free radicals primarily react with polyunsaturated fatty acids in the cell membranes, leading to lipid peroxidation. The level of lipid peroxidation significantly increases in smokers and during acute exacerbations of COPD and is known to be correlated with airflow limitations [58].

### 3.2. Protease–Antiprotease Imbalance

Elastin, which maintains elasticity in the pulmonary tissue, is degraded by proteolytic enzymes, and the elastic recoil pressure decreases, leading to the diminished elasticity of the lung tissue and resulting in emphysema [59,60]. Gottlieb et al. showed an increase in desmosine degradation from elastin excretion in the urine of patients with COPD who smoked, which is proportionally associated with a decrease in FEV1 [61]. These observations indicate that elastin degradation, resulting from an imbalance between proteolytic and anti-proteolytic enzymes, is associated with COPD pathogenesis [62]. In patients with COPD, the excessive infiltration and activation of neutrophils and pulmonary macrophages mediate the abundant secretion of proteolytic enzymes, thereby supporting the association between elastin degradation and COPD [63,64]. Moreover, the deficiency of the anti-protease α1-antitrypsin is closely correlated with the pathogenesis of COPD [65]. The deficiency of α1-antitrypsin is associated with genetic factors, attributed to the inheritance of two deficient alleles of protease inhibitor genes located on the chromosomal segment 14q32.1 [66]. Chapman et al. demonstrated that early-onset emphysema and emphysematous diseases increased in α1-antitrypsin-deficient patients, supporting the association between α1-antitrypsin deficiency and the pathogenesis of COPD [67]. In addition to elastase, matrix metalloproteinases (MMPs) are known as major contributors to COPD; in particular, levels of MMP9 are increased in the BALF of patients with COPD, and its activity is also elevated in the lung parenchyma of patients with emphysema [68]. The increase in MMP9 is known to activate tumor growth factor (TGF) β, promoting fibrosis in the airways and inducing the destruction of lung tissue [69]. Recent studies have shown that CS exposure leads to increased levels of MMP9 in the sputum and plasma of patients, resulting in the development of emphysema and decreased FEV1, carbon monoxide diffusion capacity, and oxygen saturation during COPD exacerbation [70,71]. Moreover, MMP9 expression and activity were significantly upregulated in the alveolar macrophages of patients with COPD, while they were decreased in response to metallopeptidase inhibitor 1 (TIMP1), supporting the proteolytic destruction of the lung parenchyma by proteolytic enzymes [72]. Similarly, MMP12-deficient mice exhibited the suppression of the pulmonary influx of macrophages and attenuation of emphysema development after prolonged exposure to CS [73].

### 3.3. Inflammatory Mediators

Various inflammatory mediators recruit inflammatory cells to inflammatory lesions or vasculature and trigger inflammatory responses, leading to structural changes in the lungs. In the serum of patients with COPD, cytokines such as IL6, IL8, TNFα, and IL1β are elevated, which activate immune cells including neutrophils, macrophages, and lymphocytes, contributing to the initiation and development of inflammatory responses [1,2]. These cytokines are associated with muscle weakness, cachexia, hypoxemia, and mucus hypersecretion [74]. The representative acute phase reactant, C-reactive protein, is associated with an increased risk of mortality in patients with mild-to-moderate COPD, and its elevated levels even two weeks after acute exacerbation are associated with the recurrence of acute exacerbations [75,76]. Serum amyloid A is also known to be correlated with the severity of acute exacerbations and is exhibited to be involved in the inflammatory response by activating nuclear factor kappa B (NFκB) through toll-like receptor (TLR) 2 signaling activation [77,78]. Furthermore, high levels of fibrinogen are found in patients with frequent acute exacerbations and are associated with low levels of FEV1 and an increased risk of hospitalization [79].

### 3.4. Inflammatory Cells

In respiratory inflammation, the number of activated macrophages increases in the peripheral airways, pulmonary parenchyma, and pulmonary vasculature and are accompanied by the recruitment of activated neutrophils and lymphocytes, such as cytotoxic T cell type 1, T helper type (Th) 1, and Th17 cells [80]. Additionally, in cases associated with asthma, there are instances where cells such as eosinophils, Th2 cells, or type 2 innate lymphoid cells are increased clinically [81]. These cells, along with neutrophils and macrophages, secrete inflammatory mediators and influence cells responsible for maintaining the structure of the airways, lung parenchyma, and pulmonary vasculature [1].

A prominent feature of patients with COPD is the increased activation of neutrophils and macrophages [63,64]. The number of neutrophils is increased 5–10 times in the airways, lung parenchyma, and BALF of patients with COPD compared to healthy individuals, with the number of neutrophils correlating with disease severity [82,83]. In particular, neutrophil elastase induces the degradation of extracellular matrix proteins, elastin, and collagen structures constituting the pulmonary interstitium, and exhibits resistance to α1-antitrypsin, ultimately leading to lung structural remodeling and inflammatory airway obstruction [84]. Furthermore, there is an association between increased numbers of neutrophils and decreased FEV1, airflow limitation, and a decline in the pulmonary function rate, indicating the significant involvement of neutrophils in the pathogenesis of COPD [85,86].

Activated macrophages produce inflammatory mediators such as TNFα, CXCL1, IL8, and CCL2, as well as elastolytic enzymes including MMP2, MMP9, MMP12, and neutrophil elastase, along with reactive oxygen species (ROS), leading to tissue damage in the lungs [63,64]. The characteristics of pulmonary macrophages are diversely expressed in their plasticity and are categorized into subgroups with distinct phenotypes and functions through polarization [87]. Identifying the subgroups of pulmonary macrophages that mediate innate host defense, tissue remodeling, the infiltration of inflammatory cells, and various immune responses, along with understanding the polarization mechanisms or related signaling pathways regulating them, is crucial for understanding the pathology of COPD and its prevention and treatment.

## 4. Macrophages and Their Polarization

Macrophages are immune cells that are abundant in various organs and tissues and participate in both innate and adaptive immunity, including innate host defense, the generation of inflammatory mediators, cell apoptosis, and inflammatory responses [88]. Macrophages play a crucial role in maintaining tissue homeostasis and contributing to tissue repair [89]. The characteristic feature of macrophages, such as Kupffer cells in the liver, microglia in the brain, osteoclasts in the bone, and alveolar macrophages in the lungs, lies in their diversity, exhibiting tissue-specific and functionally distinct phenotypes depending on the differentiation conditions [90,91]. Studies aimed at elucidating the origin and differentiation conditions of tissue-specific macrophages have revealed that bone marrow-derived hematopoietic stem cells (HSCs) generate circulating monocytes that subsequently differentiate into macrophages in tissues [92,93]. Recent studies have shown that in the absence of HSCs, the embryo yolk sac supplies a specific subgroup of macrophages capable of differentiating into the liver, skin, and central nervous system in mice [87]. Furthermore, it has been confirmed that tissue-resident macrophages in adults originate from macrophages that have infiltrated tissues during early embryonic stages, and not from monocytes [91].

Another characteristic of macrophages is their plasticity, which allows their differentiation into various subtypes with diverse phenotypes and functions in response to the surrounding microenvironments, tissue-derived factors, and multiple signaling stimuli [90]. The polarization of macrophages, which involves changes in their phenotypes and function in response to external signals such as growth factors and cytokines, is regulated by transcriptional regulation and external stimuli and is characterized by the expression of specific genetic markers among different phenotypes [94]. Macrophage polarization has traditionally been classified, based on in vitro studies using mouse peritoneal macrophages or bone marrow-derived macrophages (BMDMs), into two major types, M1 and M2 [90].

M1 macrophages typically exhibit pro-inflammatory polarization, which is predominantly induced by growth factors such as the granulocyte–macrophage colony-stimulating factor (GM-CSF) and inflammatory cytokines including IFNγ, TNFα, or LPS stimulation through TLR or IL1R signaling pathways, and accompanied by immune responses against bacteria and pathogens [95,96]. Regulatory factors of M1 polarization include signal transducer and activator of transcription (STAT) 1, interferon-regulatory factor (IRF) 5, and NFκB, with major downstream transcription factors such as the kruppel-like family of transcription factors (KLF) 6 and AKT2 involved [90,94]. Typically used as hallmark genes for M1 macrophages are pro-inflammatory cytokines and chemokines such as IL1α, IL1β, IL6, TNFα, CXCL2, and nitric oxide synthase 2 [90].

**Figure 4:**
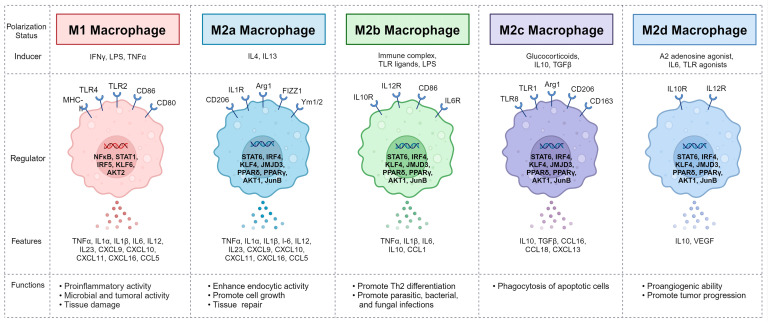
Schematic representation of macrophage polarization and their characteristics. M1 and M2 (classified into subtypes with M2a, M2b, M2c, and M2d) macrophages are activated in different ways and respond according to the stimuli represented in the figure. M1 macrophages are driven by exposure to pro-inflammatory stimuli and result in contributing to inflammatory functions. In contrast, M2 macrophages are driven by anti-inflammatory cytokines thereby, contributing to both pro- or anti-inflammatory functions and tissue repair. Created with BioRender.com. Last accessed on 10 April 2024.

M2 polarization, referred to as the alternative activation, typically exhibits anti-inflammatory and tissue repair functions and is induced by growth factors, such as the macrophage colony-stimulating factor (M-CSF) and stimulation with IL4 and IL13 [94,95]. M2 polarization is modulated by STAT6, IRF4, peroxisome proliferator-activated receptors (PPARs), and jumonji domain-containing protein-3, with downstream transcriptional regulators including KLF4, AKT1, and JunB [90,95]. M2 macrophages are characterized by the expression of surface markers such as ARG1, RETNLA, CHIL3, MGL2, MRC1, and CLEC7A [90,94]. Additionally, M2 macrophages can be classified into subtypes with distinct phenotypes, known as M2a, M2b, M2c, and M2d, in response to specific stimuli. M2a macrophages typically exhibit the M2 phenotype and display anti-inflammatory functions [91,97]. Additionally, M2a macrophages contribute to tissue repair by generating fibrosis-promoting factors such as fibronectin, TGFβ, and insulin-like growth factor [97]. M2b macrophages polarize in response to IL1R ligands or immune complexes and are characterized by reduced IL12 production and increased IL10 production. M2b macrophages not only produce IL10, but also inflammatory cytokines such as IL1β, IL6, and TNFα, contributing to immune and inflammatory responses and promoting cancer development [97]. M2c macrophages are induced by glucocorticoids, TGFβ, and IL10, exhibiting potent anti-inflammatory functions and featuring enhanced phagocytic ability by overexpressing Mer receptor tyrosine kinase (MERTK) [97]. M2d macrophages, also known as tumor-associated macrophages, undergo polarization through stimulation with IL6 or the co-stimulation of TLR ligands with the A2 adenosine receptor, featuring the overexpression of the vascular endothelial growth factor, TGFβ, and IL10. M2d macrophages have been well known to contribute to angiogenesis and tumorigenesis in tumor tissues [97]. Classified macrophage polarization and its characteristics are represented in Figure 4.

The classification of macrophages based on M1 and M2 phenotypes has been effective in functionally describing immune responses in acute infections, asthma, and obesity [98]. However, in macrophages associated with chronic inflammation and cancer, there is a limitation in adequately explaining the diverse phenotypes and their functional changes owing to the presence of more sophisticated and broad transcriptional repertoires. Recent studies have elucidated a spectral model of human macrophage activation using transcriptome-based network analyses [98]. This study revealed that the STAT4-related transcription program is activated by TNFα, prostaglandin E2 (PGE2), and P3C, while NFκB1, JunB, and cAMP response element-binding protein 1 are identified as key transcription factors in macrophage activation [98]. Additionally, it identified the absence of major inflammatory signatures in alveolar macrophages derived from patients with COPD [98]. A better understanding of the macrophage transcriptional regulation is, therefore, beneficial for the discovery of preventive and therapeutic approaches targeting multifactorial complex diseases such as COPD.

## 5. Pulmonary Macrophage

Pulmonary macrophages can be classified into two phenotypes, alveolar macrophages (AM) and interstitial macrophages (IM) [88]. AMs reside within the airway lumen and alveolar spaces, where they interact with the airway environment and epithelium, catabolize lung surfactants, perform phagocytic functions, and regulate immune responses to airborne microbes [87,91]. AMs mediate phagocytosis by recognizing microbial carbohydrates and highly expressed CD206, a marker not expressed in blood monocytes, whereas the typical monocyte surface marker, CD14, is expressed at relatively low levels [87]. Moreover, AMs are characterized by the high expression of CD11c and low expression of CD11b, distinguishing them from macrophages present in other tissue compartments [99]. IMs reside in the pulmonary interstitium, which is the space between the pulmonary epithelium and vasculature, where they engage in tissue remodeling and perform barrier immunity through antigen presentation [91,100]. IMs (CD206^+^CD169^−^) are distinct from AMs (CD206^+^CD169^+^) in that they lack the cell adhesion molecule CD169 [101]. Furthermore, IMs express high levels of CD11b and low levels of CD11c [99].

Macrophages are generally known to originate from blood mononuclear cells derived from HSC in the bone marrow or from embryonic precursors [87,92,93]. Recent studies have shown that AMs originate from fetal blood monocytes within the first week of life via a GM-CSF-dependent mechanism and are maintained independently of circulating monocytes, relying on the local self-renewal capacity [102,103]. Mature AMs persist long term in both humans and mice in the absence of inflammatory insults [104]. In contrast, IMs primarily originate from circulating monocytes derived from adult hematopoiesis [105,106]. In contrast to laboratory mice bred in specific pathogen-free environments, humans are continually exposed to respiratory pathogens, thus the ontogeny of pulmonary macrophages remains poorly understood [107]. According to lung transplantation studies, human alveolar and interstitial macrophages arise primarily from circulating monocytes and the recruited hematopoietic precursors [108,109,110]. A recent study showed that CD14^+^CD16^−^ blood monocytes have been identified as the primary precursors not only for AM and IM in humans, but also for extravascular lung monocytes derived from hematopoietic stem and progenitor cells [107]. Extravasated CD14^+^ monocytes contribute to the generation of alveolar and interstitial macrophages, whereas pulmonary intravascular macrophages have been confirmed to originate from CD16^+^ blood monocytes [107].

Compared to IM, AMs constitute over 90% of the pulmonary macrophage population [104,111]. In pulmonary design-based stereology studies, it was found that 95% of AMs reside within the diffusing airspaces, with only 5% present in the airways, whereas 78% of IMs are distributed in the alveolar septum, 14% around blood vessels, and 7% in the airways [112]. The local proliferation of AMs is primarily dependent on GM-CSF, and GM-CSF-deficient mice have shown multiple adverse effects in AMs including phagocytosis, cell adhesion, surface receptor expression (TLR and mannose receptor), and surfactant catabolism mediated by the PU.1 transcription factor, resulting in alveolar proteinosis and increased susceptibility to respiratory infections [102]. These observations are consistent with the unique airway environment associated with high oxygen tension and elevated GM-CSF levels [103]. In the steady state, AMs activate latent TGFβ via the mediation of epithelial-restricted αvβ6 integrin expression, closely associated with pulmonary epithelial cells [113]. Activated TGFβ suppresses the production of inflammatory cytokines, maintains homeostasis between the alveolar lumen and the external environment, and inhibits macrophage activation associated with pulmonary wall destruction [103,113]. However, when TLR recognizes external antigens through pathogen-associated molecular patterns, rapid actin polymerization is induced in AMs, which reactivates AM by inhibiting integrin molecule expression and suppressing TGFβ inactivation and SMAD 2 and 3 phosphorylation [103]. In the activated AM, PGE2-dependent protein kinase A signaling mediates subsequent MMP9 production and activates latent TGFβ, inducing the expression of αvβ6 integrin again, ultimately leading to the restoration of AM to its quiescent state [103].

## 6. Role of Macrophages and Their Polarization in COPD

Macrophages residing in the conducting airways and alveolar spaces play an essential role in the pathophysiology of COPD. The number of airway macrophages is closely correlated with the severity of COPD and is supported by a significant increase in macrophage numbers by approximately 5–10-fold in the airways, lung parenchyma, BALF, and sputum of patients with COPD [83,114]. Furthermore, patients with emphysema exhibited a rapid increase in the number of alveolar macrophages in the lung parenchyma when the emphysematous lesion exceeded 30% of the lung volume, and the tissue and alveolar space macrophage numbers were approximately 15.8% and 14.8%, respectively, compared to smokers with normal lung function [115]. Similarly, in smokers compared to non-smokers, the total number and local density of IM increased by 36% and 56%, respectively, in the alveolar septa [112]. These findings provide evidence of a close association between macrophages and COPD pathophysiology indicating their involvement in pulmonary tissue destruction and emphysema [1]. The pathology of emphysema is strongly associated with an imbalance between proteinases and antiproteinases, as well as the overexpression of proteolytic enzymes [116]. AMs respond to external stimuli such as CS, PM, and infections by secreting elastolytic enzymes, including MMP2, MMP9, MMP12, and cathepsin K, L, and S, which contribute to elastin and ECM degradation and the amplification of immune responses, thereby contributing to the destruction of lung parenchyma [117,118]. Compared to healthy controls, in patients with emphysema, AMs exhibit an increased production of matrix-degrading enzymes encompassing both collagenolytic and elastolytic activities in the BALF, along with increased MMP9 activity in the lung parenchyma, supporting the above observations [119,120]. A recent study has shown that an imbalance between MMPs and TIMP1 promotes ECM degradation [72]. Specifically, AMs derived from patients with COPD exhibited significantly higher MMP9 activity compared to those derived from non-smokers and healthy smokers, with decreased TIMP1 production [72].

The increased permeability of epithelial cells is closely related to respiratory tract infection, and AM is present in the airway lumen and plays an important role in respiratory tract infections caused by *Mycobacterium tuberculosis*, *Streptococcus pneumoniae*, rhinovirus, and influenza because of its ability to recognize external antigens [121,122,123]. During the early stages of infection, macrophages promote the production of pro-inflammatory cytokines and inducible nitric oxide synthase through M1 polarization, facilitating phagocytic activity [91]. Although the tissue response is designed to control infections in immunocompromised patients, it can lead to persistent infections and tissue damage. The *Mycobacterium* infection impairs phagolysosome maturation and leads to granuloma formation in infected macrophages [91]. Despite a significant increase in the number of macrophages in the lungs of patients with COPD, deficiencies in bacterial phagocytic activity have been associated with increased colonization by bacteria such as *Haemophilus influenzae*, *S. pneumoniae*, and *Moraxella catarrhalis* [124]. Similarly, patients with COPD are highly susceptible to exacerbations triggered by bacterial colonization as well as viral and bacterial respiratory infections [125]. Additionally, diminished phagocytic activity against *H. influenzae*, *S. pneumoniae*, and *M. catarrhalis* has been reported in the AMs of patients with COPD [91]. Interestingly, lower airway bacterial colonization in patients with COPD is known to increase the exacerbation frequency and is associated with a declined pulmonary function [126]. Singhet et al. showed that the impaired phagocytic activity of human monocyte-derived macrophages against *H. influenzae* was associated with an increased exacerbation frequency and disease progression in patients with COPD [127]. Recent studies have revealed that the inhibition of sphingosine 1-phosphate (S1P) signaling pathways, which mediate the maintenance of phagocytic activity in macrophages, suppresses the phagocytic ability of AMs [128]. Additionally, the overexpression of S1P receptors and S1P receptor-degrading enzymes leads to impaired phagocytic activity in AMs [128]. These findings suggest that improving macrophage dysregulation by regulating specific genes that mediate macrophage phagocytic activity could be beneficial for the prevention and treatment of respiratory tract infections.

In patients with COPD, the activation of the pro-inflammatory transcription factor NFκB in macrophages by CS or other irritants leads to the increased expression of chemokines such as IL8, CXCL1, CXCL6, and CXCL8, promoting the influx of neutrophils into the lungs and resulting in neutrophilic inflammation [117,118]. Moreover, activated macrophages induce the overexpression of CCL2 and CXCR2, thereby increasing the recruitment of monocytes and macrophages to the lungs and enhancing inflammatory responses [129]. Consistent with these observations, the increased expression of CCL2, CXCR2, and CXCL1 was observed in the sputum and bronchoalveolar lavage (BAL) of patients with COPD [129]. AMs from patients with COPD and mice chronically exposed to CS overexpressed CLEC5A, exacerbating the expression of inflammatory cytokines and airway inflammation [130]. In another study, a deficiency of isthmin 1 (ISM1) in mice led to an increase in the functional heterogeneity and recruitment of AMs, resulting in enhanced pulmonary inflammatory responses, emphysema, and impaired lung function [131]. Furthermore, ISM1 inhibits MMP12 production and inflammatory responses by inducing the apoptosis of the cell-surface G-protein-coupled receptor 78^high^ AM, thereby contributing to the maintenance of pulmonary homeostasis [131]. A recent study revealed that in mice deficient in nucleotide-binding oligomerization domain-like receptor 3 (NLRP3), pulmonary function improves, clinical manifestations decrease, and there is a reduced recruitment of macrophages along with a decreased production of inflammatory cytokines [132]. Additionally, Wang et al. showed that the mRNA levels of genes constituting the NLRP3 inflammasome complex were significantly higher in patients with exacerbated COPD than in smokers and that the NLRP3 inflammasome in the lower respiratory tract was positively correlated with the bacterial burden in smokers and patients with COPD [133].

In addition to inflammation, oxidative stress in COPD pathogenesis induces mitochondrial dysfunction, the overproduction of TGFβ and nitric oxide, and notably, leads to the impaired phagocytic function of alveolar macrophages [58]. A recent study showed that AMs and monocyte-derived macrophages (MDM) from patients with COPD exhibited mitochondrial respiration- and glycolysis-mediated metabolic energy exhaustion and compromised redox homeostasis [134]. However, such a functional decline and abnormalities in AMs and MDMs can be restored through the activation of the nuclear factor erythroid 2–related factor 2 (NRF2) pathway [134]. Similarly, oxidative stress induced by mitochondrial ROS in BAL-derived AMs from patients with COPD has been shown to diminish the phagocytic activity of AMs against infections [135].

Macrophage polarization in patients with COPD is highly diverse and heterogeneous. Eapen et al. showed that functionally undifferentiated M0 macrophages predominate in the normal airway epithelium with very low levels of M1 macrophages present, whereas in the subepithelium, M1 and M2 macrophages are predominant, comprising 1.6% and 36%, respectively [136]. Patients with COPD exhibited 21.2% M1 macrophages in the airway epithelium, with a minimal presence of M2 macrophages, whereas in the subepithelium, M1 and M2 macrophages were observed at 9.6% and 8.4%, respectively [136]. Furthermore, an examination of the M1/M2 pattern of AMs derived from the BAL lumen revealed that in the control group, M1 macrophages predominated at a ratio of 66.3%, with a lower proportion of M2 macrophages. However, in patients with COPD, the M1 phenotype decreased to 40.4%, and the M2 phenotype increased to 27.15% [136]. Additionally, BAL cytokine production in patients with COPD augmented the M2 profile, including CCL22, IL4, IL13, and IL10 [136]. Enhanced M2 profiles in COPD reflect a mixed pattern of contradictory aspects. M2 profiles in COPD promote the production of proteolytic enzymes, such as MMP9 and MMP12, inducing the activation of mucin-related genes and contributing to lung tissue damage, emphysema, and the development of airway obstruction, while inducing wound repair and inflammation resolution [99]. These findings suggest that the polarization of macrophages in COPD varies according to the microenvironment of different tissues and is closely associated with the cytokine profile in each tissue. Recent RNA sequencing studies indicate that the typical classification of M1/M2 polarity in COPD is inadequate to explain the diverse phenotypes and functions of macrophage subsets within the intricate microenvironment of the lungs [137]. Takiguchi et al. demonstrated that approximately 25% of BALF-derived double-negative macrophages (CD40^−^CD143^−^), relatively abundant in COPD, exhibited pro-inflammatory properties and the functional impairment of cellular homeostasis, which may contribute to the pathogenesis of inflammatory disorders [137]. Another study hypothesized that patients with human immunodeficiency virus (HIV) infection are at an increased risk of developing chronic inflammatory diseases such as COPD due to immunodeficiency. In this study, the phenotype of AMs in HIV patients with COPD was characterized into four subgroups classified as M1 (CD40^+^CD163^−^), M2 (CD40^−^CD163^+^), double positives (CD40^+^CD163^+^), and double negatives (CD40^−^CD163^−^) [138]. In addition, the number of double negative macrophages was significantly reduced to 84.1% in HIV patients with COPD, and to 54.3% and 23.9% in HIV patients without COPD and normal subjects, respectively, and the phagocytic ability was significantly reduced in this phenotype [138].

CS is a major risk factor for COPD, contributes to the onset and progression of inflammatory responses, and affects macrophage function and phenotypes [139]. Typically, CS exposure induces inflammatory gene expression such as IL8 and TNFα through the NFκB pathway in macrophages [140,141]. However, several studies have shown that various M2 polarizations were increased, while M1-polarized phenotypes were decreased in AMs following CS exposure [142,143,144]. Shaykhiev et al. showed that inflammatory and immune response-related gene expression, including M1 profiling, was suppressed in AM from smokers [145]. Furthermore, the transcriptional profiling of AM exhibited that CS exposure induces significantly up-regulated tissue remodeling and immunoregulatory-associated genes including MMP2, MMP7, and adenosine A3 receptor (ADORA3) [145]. Similarly, mycobacterial containment and inflammatory gene expression, such as IFNγ and TNFα, diminished in AMs following CS exposure [146]. Interestingly, a recent study demonstrated that altered M2 polarization following CS exposure was restored by smoking cessation in the murine AMs [147].

Taken together, pulmonary macrophages play a pivotal role in various pathological processes including inflammatory and immune responses, phagocytosis, and the maintenance of either tissue or oxidative/reductive homeostasis in COPD, based on their diversity and plasticity. Therefore, it is important to understand the mechanisms associated with macrophages. Furthermore, understanding and categorizing the diverse differentiated macrophage phenotypes induced by the surrounding microenvironment and identifying their specific functions are expected to aid in comprehending the pathogenesis of COPD and formulating preventive and therapeutic strategies.

## 7. Altered Function of Macrophage in Patients with COPD

Macrophages are the most predominant immune cells present in BALF and lungs. Several studies have suggested that alveolar macrophages were increased in the BALF or lung tissue of patients with COPD compared to control subjects [148,149], although a study reported that there was no significant increase in alveolar macrophages in the BALF of patients with COPD [150]. The increased number of macrophages in COPD may result from delayed apoptosis [151]. Moreover, the number of alveolar macrophages was higher in GOLD 3–4 than in GOLD 1–2 patients, implying a positive correlation between the number of macrophages and the severity of COPD [152]. Preclinical models of COPD also have shown increased levels of macrophages in the BALF and lung tissues, with similarity to clinical results [153,154]. AMs play a crucial role in the maintenance of homeostasis, pathogen clearance, and the resolution of inflammation in the lungs. In addition to increased macrophage numbers, functional changes have been documented in COPD patients, notably impaired phagocytosis, phenotype shifts, inflammatory responses, and lipid-laden macrophages in patients with COPD.

### 7.1. Altered Macrophage Phenotype

Macrophages are categorized into M1, M2, dual-polarized, and non-polarized. Emerging research has reported that patients with COPD have different macrophage phenotypes compared to non-COPD subjects in the BALF and lungs, as described in Table 1. Two independent clinical studies have found a significant increase in the proportion of non-polarized macrophages (CD40^−^CD163^−^) in the BALF from patients with COPD [137,155]. However, another study involving healthy controls, smokers, COPD-current smokers, and COPD-ex smokers revealed no significant changes in the population of non-polarized M0 macrophages (CD68^+^iNOS^−^CD163^−^) among the groups [136], but the percentage of iNOS^+^ M1 macrophages was decreased in the smokers and patients with COPD, while CD163^+^ M2 macrophages were increased in the smokers and patients with COPD compared to healthy controls. In addition, AM represents higher levels of M2-related genes such as ADORA3, MMP2, and MMP7, whereas M1-related genes such as CXCL9, CXCL10, CXCL11, and GBP5 were decreased in the COPD smokers compared to healthy non-smokers [145]. Meanwhile, the percentages of CD86^+^ M1 and CD206^+^ M2 macrophages were significantly increased in patients with COPD; in particular, M1 macrophages were significantly associated with COPD severity [152]. These inconsistent observations may be due to variations in the methods of macrophage analysis, different macrophage markers, or characteristics of patients. Therefore, classifying and analyzing the contradictory phenotype and function of macrophages in COPD, considering variables such as smoking history, age, and severity of COPD, can be beneficial to better understanding the role of macrophages contributing to the diverse pathogenesis of COPD.

The severity of COPD is associated with the macrophage phenotype. Specifically, the percentage of non-polarized macrophages (CD40^−^CD163^−^) in the BALF was positively correlated with COPD severity [155]. In addition, non-polarized macrophages show decreased phagocytic activity against *Staphylococcus aureus*, explaining the impaired phagocytosis in patients with COPD. Moreover, non-polarized macrophages exhibit reduced gene expression for pathogen recognition and processing (CD163, CD40, CCL13, and C1QA and B) and increased gene expression for inflammation promotion (CXCR4, RAF1, amphiregulin, and MAP3K5). Furthermore, smoking also can affect the macrophage polarization in patients with COPD. COPD smokers show a significantly lower percentage but a higher number of CD163^+^ M2 macrophages than COPD ex-smokers in the BALF, but not sputum [159].

The macrophage phenotype in the lungs of patients with COPD also exhibited differences between COPD patients and controls. Patients with COPD and smokers showed increased levels of iNOS^+^ M1, CD206^+^ M2, and iNOS^+^CD206^+^ dual-polarized macrophages compared to the controls, with a progressive increase in the severity of COPD with dual-polarized macrophages [157]. Conversely, another study revealed that increased numbers of M2 macrophages are associated with COPD severity. GOLD 3–4 exhibit significantly higher numbers and percentages of M2 macrophages (CD163^+^, CD204^+^, or CD206^+^) than GOLD stages 1–2, smokers, and non-smokers [158]. In addition, the number of M2 macrophages was negatively correlated with lung function, as evidenced by an association between the increased numbers of M2 macrophages and a reduced percentage of FEV1. The M1 macrophage and related genes including IL1β, TLR2, TLR4, and CD86 were found to be elevated in the lung and tracheal tissue samples of patients with COPD compared to healthy controls [160]. Despite some inconsistencies in the results, these findings suggested that COPD patients have distinct macrophage phenotypes with non-COPD subjects, thereby indicating the potential efficacy of regulating macrophage phenotype alterations as a therapeutic option for COPD treatment.

### 7.2. Impaired Phagocytic Activity

Phagocytosis is the process of binding to recognition receptors and ingesting particles exceeding 0.5 μm, including pathogens, apoptotic cells, and foreign particles, playing a pivotal role in the innate immune system. Macrophages are key effectors in the resolution of inflammation through phagocytosis and efferocytosis, the phagocytosis of apoptotic cells, to prevent the release of pro-inflammatory and toxic factors from dying cells. The phagocytosis of AM is crucial in sterile lung conditions because it removes pathogens. Impaired phagocytosis has been observed in various respiratory diseases, including asthma, cystic fibrosis, idiopathic pulmonary fibrosis, and COPD. Poor phagocytic activity may lead to lower airway bacterial colonization, which is a common condition observed in approximately half of all patients with COPD. The predominant bacterial infections in patients with COPD are caused by *H. influenzae*, *M. catarrhalis*, and *S. pneumoniae* [161].

AMs from COPD patients have a decreased phagocytic activity compared to controls challenged with various pathogens, including *E.coli* [162], *S. pneumonia* [163], and non-typeable *H. influenzae* (NTHI) [163,164], as well as the efferocytosis of apoptotic cells [163,165]. AMs of both COPD ex-smokers and active smokers showed the impaired phagocytosis of NTHI and *M. catarrhalis,* but not *S. pneumoniae* or microspheres [164]. Furthermore, phagocytic activity against *E. coli* is decreased in both AM and MDM from COPD patients [166]. MDM is frequently utilized as a substitute for AM owing to its similar characteristics. In addition, the MDM phagocytosis of *H. influenzae* and *S. pneumoniae* was also decreased in patients with COPD. Bacterial colonization is related to the exacerbation of COPD and accelerates lung function decline. MDM from COPD’s frequent exacerbations showed the decreased phagocytosis of *H. influenzae* but not *S. pneumoniae* [127]. The impaired COPD macrophage phagocytosis of *H. influenzae* is associated with the exacerbation frequency, resulting in pro-inflammatory macrophages that may contribute to disease progression.

Efferocytosis involves the clearance of apoptotic cells to resolve inflammation. Impaired phagocytosis could induce the accumulation of apoptotic cells, leading to secondary necrosis inflammation and tissue damage. AM from patients with COPD showed an impaired efferocytosis ability, showing a negative correlation between the efferocytosis of AM and mRNA levels of *SPHK1*, *S1PR3*, and *S1PR5* [165]. In addition, AMs and MDMs in patients with COPD have an impaired ability of efferocytosis compared to non-COPD subjects [163,167].

Impaired phagocytosis is linked to the reduced expression of phagocytic receptors [168]. AMs from COPD patients exhibited decreased percentages and levels of phagocytic receptors, such as Fcγ receptor I (FcγRI), macrophage scavenger receptor 1 (MSR1), and Siglec-1 compared to never smokers. Specifically, Siglec-1 is significantly lower in COPD patients than ex-smokers, and the use of the anti-Siglec 1 blocking antibody decreased the phagocytosis of NTHI [169]. These findings suggest the importance of the improvement of phagocytosis or efferocytosis by the modulation of phagocytic receptors.

### 7.3. Inflammatory Response

Several studies have demonstrated the distinct inflammatory response of AMs cultured from BALF in patients with COPD compared to control subjects. Culpitt et al. reported the increased secretion of IL8 in COPD AMs under basal conditions, with no significant changes in IL8 and GM-CSF in response to LPS stimulation [170]. Conversely, another study found that basal IL8 release showed no difference between patients with COPD and controls, with increased levels of IL8 and MMP9 and decreased levels of GM-CSF and IL6 upon LPS stimulation in COPD AMs compared to controls [171]. Additionally, TGFβ and TIMP1 are released less from the AMs of patients with COPD following LPS stimulation [172]. Higham et al. reported no significant changes in TNFα, IL6, and CXCL8 with or without LPS stimulation [173].

SIRT1 is known to play an important role in anti-inflammatory, anti-aging, and DNA repair activities and is decreased in several disease conditions. SIRT1 deacetylates the nuclear p65 subunit of the transcription factor NFκB to suppress NFκB-dependent pro-inflammatory gene transcription. Patients with COPD also showed lower levels of SIRT1 in lung tissue and lung macrophages [174]. SIRT1 is decreased and RelA/p65 NFκB is increased in the macrophages in the lungs of patients with COPD compared with non-COPD subjects; the diminished SIRT1 could lead to impaired anti-inflammatory action [174]. In addition, the single-cell RNA sequencing of human lung tissues showed that chemotaxis and inflammation are increased in the alveolar macrophages of patients with COPD, as evidenced by changes in THBS, PELI1, and CDC42 [175]. Further studies are needed to elucidate the inflammatory response in COPD patients.

### 7.4. Lipid-Laden Foamy Macrophages

Foam cells, also called lipid-laden cells, are a type of cell that contains cholesterol, which is formed by an increased lipid uptake or altered lipid metabolism. Foam cells are highly present in chronic inflammation in many infectious and metabolic diseases, and some cancers. Concurrently, critical macrophage immune functions are diminished. Lipid-laden alveolar macrophages or pulmonary foamy macrophages are known to have impaired phagocytosis, more programed cell death, and inflammation. Lipid-laden macrophages are identified in several pulmonary diseases including silicosis, pulmonary alveolar proteinosis, tuberculosis, and COPD [176,177,178,179].

The BALF from COPD patients showed an increased number of lipid-laden foamy macrophages [179]. Compass analysis predicted that an altered lipid metabolism and metabolism-associated genes (CD36, COLEC12, SOAT1, and PPARγ) were upregulated in the BALF-derived macrophages from patients with COPD. In addition, macrophages from patients with COPD show a significantly elevated lipid class of cholesteryl esters, suggesting the presence of foam cell-like macrophages [180]. Another study showed that CD1b, a lipid antigen-presenting molecule, was significantly increased in the AMs of patients with COPD and correlated with lung function (FEV1) and the smoking pact year history [181]. However, another transcriptome and single-cell analysis showed the downregulated CD1b expression in AMs [150]. The transcriptome and lipidome analysis of AMs from patients with COPD showed GOLD grade-associated changes, which are lipid-related gene sets including the fatty acid catabolic process, fatty acid oxidation, and the regulation of the cholesterol biosynthetic process in GOLD 2 COPD patients, with an enrichment in the cholesterol and lipid storage in GOLD 3–4 compared to the control [150].

## 8. Macrophage-Targeting COPD Treatment

Macrophage dysregulation is closely associated with the pathology and severity of COPD, thus restoring the macrophage phenotype, impaired phagocytosis, increased inflammation, and elevated foamy macrophage could be a promising target for treatment for COPD patients (Table 2).

### 8.1. Regulation of Macrophage Phenotype

Emerging preclinical studies have revealed macrophage polarization dynamics in COPD models. Several COPD preclinical models showed elevated levels of M1 macrophages, with a reversal of this increase attenuating lung inflammation [182]. Treatment with salidroside ameliorated pulmonary inflammation, emphysema, and the lung function, while concurrently suppressing iNOS^+^ M1 macrophage polarization in the lungs of a CS-induced lung inflammation model [182]. In addition, salidroside inhibited the percentage and protein expression of iNOS^+^ M1 macrophages in AMs by the inhibition of JNK/c-Jun. Moreover, hydrogen intervention reversed the increased levels of IL6, TNFα, and TGFβ while decreasing the levels of IL10 with an improvement in the lung function in CS exposure with LPS models [183]. Sun et al. observed that ergosterol treatment reduced the levels of M1-associated cytokines (IL6 and TNFα) while increasing M2-associated cytokines (IL10 and TGFβ) in BALF. Ergosterol treatment also decreased CD40^+^ M1 macrophages and increased the CD163^+^ M2 macrophage in the lungs of cigarette smoke extract (CSE)-induced COPD rats [184]. In addition, ergosterol decreased the CS-induced CD40^+^ M1 macrophage and increased the CD163^+^ M2 macrophage in RAW 264.7 cells. Rosiglitazone, a potent agonist for PPARγ treatment, decreased iNOS^+^ M1 macrophages in the lungs and reduced the CD86^+^ M1 macrophage percentage in CS-induced-M1 macrophage polarization, mediated by the activation of PPARγ and RXRα [185]. Furthermore, PPARγ agonist treatment attenuated the airway inflammation, decreased CD86^+^ M1 macrophages, and increased CD206^+^ M2 macrophages in the BALF, while the knockout of PPARγ exacerbated airway inflammation, increased M1 macrophages, and decreased M2 macrophages [152].

Meanwhile, M2 macrophage polarization has been shown to increase in PM2.5 [186], CS exposure [187], and LPS with CS-induced [188] COPD. MicroRNAs (miRNAs), small non-coding RNAs typically composed of 21–25 nucleotides, are implicated in COPD pathogenesis. miRNAs from AMs of patients with COPD or smokers exhibited altered expression levels [189,190]. The regulation of miRNAs has been shown to influence the macrophage phenotype. For example, the knockout of miR-21, which is increased in the lung tissue and BMDM of the COPD model, showed decreased levels of M2-related genes (CD206, ARG1, YM1, FIZZ1, and IL10) [187]. The overexpression of microRNA let-7c, which is downregulated in the lungs of patients with COPD, inhibited CSE-induced M2 polarization in vitro by the inhibition of the IL6/STAT3 pathway [156]. PM2.5 induced M2 macrophage polarization by the inhibition of histone deacetylase 2 (HDAC2). In addition, a HDAC2 deficiency leads to the aggregation of inflammation and the induction of M2 macrophage polarization [186]. The treatment of effective-components combination (ECC), comprising 20-S-ginsenoside Rh1, astragaloside IV, icariin, nobiletin, and paeonol, suppressed the CD 206^+^ M2 macrophage polarization in the lungs and attenuated airway remodeling in COPD rats. In addition, ECC treatment reduced M2 macrophage polarization induced by IL4 treatment by inhibiting mTORC2 activity in MH-S cells [191]. The regulator of telomere elongation 1 (RTEL1) expression was increased in the lung tissues of COPD mice, and the knockdown of RTEL1 increased the CD206 M2^+^ macrophages and pulmonary inflammation [188].

### 8.2. Enhanced Phagocytic Activity

Phagocytosis impairment is identified in the AMs of patients with COPD, possibly increasing the bacterial infection. Various studies were conducted to enhance the phagocytic activity of AM. Several types of antibiotics are used to treat COPD because bacterial infection is commonly observed in patients with COPD. Azithromycin, one of the macrolides, is used to treat exacerbations in patients with COPD. Following 12 weeks of azithromycin therapy, AM from patients with COPD exhibited increased efferocytosis of apoptotic bronchial epithelial cells and phagocytosis of *E. coli*, but not of beads [162,192]. In addition, azithromycin therapy increased mannose receptor expression and reduced the levels of inflammatory markers in the peripheral blood. The nonantibiotic macrolides macrolides-2′-desoxy-9-(S)-erythromycylamine (GS-459755) and azithromycin-based 2′-desoxy molecule (GS-560660) restore the CS-induced impaired phagocytosis and efferocytosis of apoptotic bronchial cells in human alveolar macrophages and THP-1 cells [193].

Corticosteroids, including dexamethasone, budesonide, and fluticasone propionate could increase the relative expression of M2-associated genes such as CD163, CD206, and MERTK while decreasing the expression of M1-associated genes such as CD64, CD80, and CD86 in macrophages from the BALF of patients with COPD [194]. Dexamethasone treatment increased the percentage of the efferocytosis of apoptotic neutrophils, but it had no impact on the percentage of phagocytosis of opsonized NTHI or *M. catarrhalis*. Another clinically used to treat COPD, roflumilast, a phosphodiesterase-4 inhibitor, increased the phagocytic activity after treatment for 3 and 6 months [195].

Apart from the treatments mentioned earlier that are used in clinics, researchers are exploring different methods to improve phagocytosis in COPD. In macrophages derived from BALF, patients with COPD showed higher levels of genes related to the S1P system compared to controls. A strong negative correlation was found between the mRNA expression levels of *SPHK1*, *S1PR3*, and *S1PR5* and the ability of AMs to phagocytose apoptotic cells as well as lung function (FEV1). The phagocytosis of apoptotic cells was increased by the addition of the S1PR3 and S1PR5 agonist suramin [165]. In addition, CSE exposure to MH-S cells decreased the phagocytic ability against *E. coli* and downregulated the relative mRNA expression levels of miR-155-5p [196]. The miR-155-5p mimic increased the phagocytosis of MH-S cells, whereas the inhibition of miR-155-5p decreased the phagocytosis by the mTORC2/RhoA pathway.

Moreover, the activation of NRF2 reversed the impaired phagocytic activity. Sulforaphane, an NRF2 activator, improved the bacterial clearance of NTHI and *Pseudomonas aeruginosa* by activating NRF2 and increasing MARCO [197]. CS exposure impairs bacterial clearance, which was worsened in an NRF2 knockout mouse, demonstrating the importance of NRF2 in phagocytosis. In addition, NRF2 agonist sulforaphane and compound 7 increased the impaired nonopsonic and opsonic phagocytosis of *S. pneumonia* [198]. Thus, the improvement of phagocytosis could be a promising target to attenuate COPD symptoms.

### 8.3. Reduced Inflammatory Response

Although numerous strategies are employed to alleviate COPD by suppressing inflammatory cascades, only a limited number of investigations were conducted on targeting the inhibition of macrophage inflammation for COPD treatment. PDE4A, B, and D are increased in the AMs and lung tissues of patients with COPD compared to the controls, except for PDE4A in the lung tissues. The PDE4 inhibitor roflumilast decreased TNFα against LPS treatment in AMs and whole lung tissue from patients with COPD [199]. Resveratrol reduced IL8 and GM-CSF in basal, IL1β-stimulated, and CS-medium conditions in AMs from COPD patients and smokers [200]. Additionally, patients with COPD showed higher levels of miR-486-5p in AMs compared to controls [190]. The miR-486-5p mimic promoted the inflammation induced by CSE, while conversely, an inhibitor of miR-486-5p decreased the inflammatory response by the inhibition of TLR4-triggered inflammation.

### 8.4. Reduced Lipid-Laden Foamy Macrophages

Restoring dysregulated expression in COPD could be one of the promising targets for COPD treatment. Surfactant protein D (SP-D), a member of the collectin family, plays a crucial role in the regulation of pulmonary surfactants and the maintenance of lipid homeostasis in the lungs. SP-D levels are decreased in the BALF of patients with COPD and animal models [192,201], whereas they are increased in the BALF and serum of ozone-induced COPD mouse models [179]. A clinical study showed that the serum levels of SP-D are increased, but the BALF levels of SP-D are decreased in patients with COPD, showing a positive correlation between SP-D levels in BALF and FVC or FEV1 [201]. In addition, the knockout of SP-D aggravated pulmonary inflammation and emphysema, accompanied by an increase in lipid-laden foamy macrophages. Conversely, exogenous SP-D treatment ameliorated lung inflammation in ozone-induced COPD and oxidized low-density lipoprotein-induced foamy macrophage formation [179]. Additionally, the relative expression of miR-103a is decreased in AMs from patients with COPD compared to non-smokers and in macrophage cells, including THP-1 cells, BMDM, and MH-S cells following CSE treatment. The overexpression of miR-103a decreased the lipid-laden macrophage formation in THP-1 cells treated with CSE and in oxidized low-density lipoprotein-treated mice [202]. Although further studies are needed to elucidate the mechanisms, the reduction of lipid-laden foamy macrophages could be one of the therapeutic strategies for COPD.

This review proposes that restoring macrophage dysfunction is a promising target for COPD management. Clinically used interventions for COPD such as macrolides and corticosteroids have shown efficacy in enhancing phagocytosis activity. However, further investigations are necessary to fully understand the impact of macrophage targeting on COPD progression.

**Table 2:** Treatments of COPD targeting macrophage function.

Therapeutic Means	Target and Mechanisms	Effect on Macrophage Function	Ref.
*Plant compound* (Salidroside)	JNK/c-Jun	Inhibited M1 polarization of alveolar macrophages in lung tissues of CS-induced pulmonary inflammation rat model and alveolar macrophage from BALF	[182]
*Mycosterol* (Ergosterol)	HDAC3	Inhibited M1 polarization and increased M2 polarization in CSE-induced COPD rat model and raw cells Elevated HDAC3 activation and suppressed HAT activity and NFκB/p65 acetylation	[184]
*Thiazolidinedione compound* (Rosiglitazone)	PPARγ and RXRα	Inhibited M1 polarization in lung and BALF of CS-induced COPD model and alveolar macrophage from BALF	[185]
PPARγ agonist	JAK-STAT, MAPK and NFκB	Inhibited M1 polarization and increased M2 polarization in CS-induced COPD mice	[152]
*Plant compound* (ECC)	mTORC2	Inhibited M2 macrophage polarization by inhibition of mTORC2 activity in IL4-induced polarization in MH-S cells	[191]
*Macrolide antibiotics* (Azithromycin)		Increased phagocytosis of *E. coli* and efferocytosis	[162,192]
*Nonantibiotic macrolides* (GS-459755, GS-560660)	NLRP3	Increased phagocytosis of NTHI and efferocytosis Decreased the NLRP3 and IL1β in THP-1 cells	[193]
*Corticosteroid* (Dexamethasone)		Increased efferocytosis	[194]
*S1PR3 and S1PR5 agonist* (Suramin)		Increased efferocytosis	[165]
*Plant compound* (Sulforaphane)	NRF2	Increased phagocytosis of NTHI and *P. aeruginosa* by increasing MARCO	[197]
*PDE4 inhibitor* (Roflumilast)		Increased phagocytosis	[195]
*PDE4 inhibitor* (CHF6001 and roflumilast)	CREB	Inhibited TNFα stimulated with LPS in alveolar macrophage and lung tissues of patients with COPD	[199]
Exogenous SP-D	NRF2	Decreased lipid-laden macrophages Improved lung function and attenuated airway inflammation in ozone-exposed mice	[179]
miR-103a		Decreased lipid-laden macrophages	[202]

## 9. Conclusions

COPD represents increases in prevalence and mortality with airflow limitation and persistent clinical symptoms, currently managed primarily through a combination of β2-agonists and long-acting muscarinic antagonists with inhaled corticosteroids following GOLD guidelines due to the complex multifactorial pathogenesis. The identification of increased levels of macrophages in patients with COPD and the clinical correlation between the broad inflammatory and immune responses and oxidative stress highlights the role of pulmonary macrophages as a potential target for COPD treatment. Regulating pulmonary macrophage-mediated functions, including the maintenance of protease and antiprotease homeostasis, phagocytic activity, and broad immune and inflammatory responses, emerge as a prospective therapeutic approach for patients with COPD. Therefore, additional research, particularly through clinical trials, and understanding the differentiated macrophages and their specific functions induced by the surrounding microenvironment is imperative for comprehensively assessing macrophage polarization as a therapeutic approach for COPD. 

## Figures and Tables

**Table 1:** Altered macrophage phenotypes in patients with COPD.

	M1	M2	DP	DN	Source	Method	Ref
Marker	CD40^+^CD163^−^	CD40^−^CD163^+^	CD40^+^CD163^+^	CD40^−^CD163^−^	BALF	Flow cytometry	[155]
COPD (vs. NC)	NS	NS	Decreased	Increased			
Marker	CD40^+^CD163^−^	CD40^−^CD163^+^	CD40^+^CD163^+^	CD40^−^CD163^−^	BALF	Flow cytometry	[137]
COPD (vs. NC)	NS	NS	Decreased	Increased			
Marker	CD86^+^	CD206^+^			BALF	Flow cytometry	[152]
COPD (vs. NC)	Increased	Increased	No detection	No detection			
Marker	iNOS^+^	CD206^+^			Lung tissues	IHC	[149,156]
COPD (vs. NC)	Decreased	Increased	No detection	No detection			
Marker	iNOS^+^	CD206^+^	iNOS^+^CD206^+^	iNOS^−^CD206^−^	Lung tissues	IHC	[157]
COPD (vs. NC)	Increased	Increased	Increased	Decreased			
Marker		CD163^+^, CD204^+^, or CD206^+^			Lung tissues	IHC	[158]
COPD (vs. NC)	No detect	Increased	No detection	No detection			

## Data Availability

Not applicable.
